# An outbreak of Salmonella Enteritidis food poisoning following consumption of chicken shawarma: A brief epidemiological investigation

**DOI:** 10.12688/f1000research.54410.2

**Published:** 2021-12-06

**Authors:** Surendran Deepanjali, Mandal Jharna, Bammigatti Chanaveerappa, Dhandapani Sarumathi, Pallam Gopichand, Kaliyappan Anupriya

**Affiliations:** 1 Department of Medicine, Jawaharlal Institute of Post Graduate Medical Education and Research, Dhanvantri Nagar, Puducherry, 605006, India; 2 Department of Microbiology, Jawaharlal Institute of Post Graduate Medical Education and Research, Dhanvantri Nagar, Puducherry, 605006, India

**Keywords:** Salmonella Enteritidis, foodborne disease outbreaks, gastroenteritis

## Abstract

Background: Shawarma, a popular meat-based fast food could be a source of foodborne outbreak due to non-typhoidal *Salmonella *(NTS). A clustering of acute gastrointestinal (GI) illness following intake of chicken shawarma occurred primarily among the staff and students of a tertiary care hospital in southern India.

Methods: A case-control study was conducted among 348 undergraduate medical students (33 cases, 315 controls).  Data was collected using direct interviews and a simple online questionnaire. Epidemiological associations of GI illness were evaluated at three levels of exposure namely-eating food from any restaurant, eating food from the implicated food outlet, eating chicken shawarma from the implicated outlet.

Results: Of 33 cases, 26 had consumed food from a particular food outlet, 4 from other outlets, and 3 did not report eating out. Consumption of food from the suspected food outlet was significantly associated with GI illness (odds ratio 121.8 [95% CI 28.41 to 522.66]; *P*<0.001); all the 26 cases who had eaten from the particular outlet had eaten chicken shawarma. By comparison, only one of the 315 controls had eaten this dish. Of the 27 persons (cases as well as controls) who had consumed chicken shawarma from the outlet, 26 were ill. Culture of stool samples from 10 affected individuals and implicated food item yielded *Salmonella* Enteritidis.

Conclusions: Meat-based shawarma is a potential source of NTS infection. Food safety authorities should enforce guidelines for safe preparation and sale of shawarmas and similar products.

## Introduction

Shawarma is a meat-based dish of Middle Eastern origin. ^1^ It has become a popular food item across many countries including India. Nontyphoidal *Salmonellae* (NTS) are known to contaminate meat and poultry products resulting in foodborne disease outbreaks. ^2^ There have been a few recent reports of foodborne disease outbreaks related to NTS contamination of chicken shawarma. ^3– 5^ A few countries have issued guidelines for safe preparation and serving of shawarma, ^6, 7^ but such guidelines do not exist in many developing countries. Further, foodborne disease outbreaks are often under-reported, and the necessary epidemiological investigations are not always carried out. ^8, 9^ Here, we report an epidemiological investigation of a foodborne disease outbreak caused by consumption of chicken shawarma, which mainly affected the students and staff of a teaching hospital.

## Methods

### Setting

This brief outbreak investigation was carried out during the months of July and August 2019 at the Jawaharlal Institute of Postgraduate Medical Education and Research, which is a tertiary care hospital in Puducherry, India. Data presented in the study was collected as part of an outbreak investigation which was done as a public health exercise to identify the source of infection and taking steps to prevent further infections. The participating subjects were aware that their data was being collected as part of a foodborne disease outbreak investigation. However, since the decision to publish the findings was taken many months after the outbreak investigation, an informed consent for publication was not explicitly taken. An exemption from review was granted by the Institute's Ethics Committee.

### Recognition of the outbreak and evaluation of affected individuals

An outbreak was suspected when several individuals were hospitalized with gastrointestinal complaints after consumption of chicken shawarma from a particular restaurant on dates July 22-24. Clinical history was collected through direct interview from individuals who were still hospitalized when data collection started and through telephonic conversation from individuals who could not be directly interviewed. The clinical history of those who were treated in pediatrics department of our institution for similar gastrointestinal complaints and also a few individuals treated elsewhere were obtained from patients who were admitted in Department of Medicine and who happened to share the chicken shawarma meal.

### Microbiological investigations

As part of the routine clinical care of admitted individuals, primary culture of stool samples was done. Since all the affected individuals had chicken shawarma, as part of outbreak investigation, a specimen of the implicated food item was obtained on the same day and subjected to culture. Cultures were done in MacConkey, XLD, DCA, TCBS with selenite F enrichment and alkaline peptone water. Identification of the bacterial colonies were done using matrix assisted laser desorption ionization-time of flight mass spectrometry (MALDI-TOF MS version 3.2, VITEK MS, Biomerieux). Stool polymerase chain reaction (PCR) was done using Eppendorf AG HAMBURG 22331 for identifying diarrheagenic *E. coli* and *Campylobacter* spp. Serotyping was done by the conventional method using polyvalent and type-specific monovalent antisera (Denka Seiken, Japan).

### Case-control study

As part of the outbreak investigation, we conducted a case-control study for confirming the source of contaminated food. Since undergraduate medical students belonging to the third to ninth semesters constituted a major proportion of affected individuals, we considered them representative of the population at risk. We collected information from them by direct interview which was carried out by meeting the third to ninth semester students when they assembled for scheduled theory classes. Those who could not be directly interviewed were requested to fill up a web-based (Google forms) questionnaire which was circulated through the respective classes’ social media groups. We asked three questions: ‘Where did you have your dinner on July 21?’ (the day prior to presentation of index case); ‘What did you eat?’; and ‘Did you have any gastrointestinal (GI) symptoms in the form of abdominal pain, vomiting or diarrhea during the index dates from July 22 to 24?’.

We defined cases as those who presented with GI symptoms on dates July 22-24 irrespective of need for hospital admission. Controls were those who reported no GI symptoms. We compared the occurrence of GI illness at 3 levels of exposure among cases and controls at 3 levels. At each level the odds ratio was calculated as ratio of odds of illness in the exposed group to the odds of illness in the non-exposed group. Level 1 was eating at any place other than their hostel or home on July 21st; Level 2 was consumption of any food item from the suspected food outlet on July 21st among those who ate outside; and Level 3 was consumption of chicken shawarma among those who had dined at the suspected outlet. To assess the causality of the observed epidemiological association, we applied the Bradford Hill's criteria adopted for foodborne disease outbreaks. ^10, 11^

## Results

The index case was a postgraduate resident who presented to the emergency department at 4 AM on July 22, 2019 with abdominal pain and multiple episodes of diarrhea which started about 7.5 hours after consuming biryani (a mixed rice dish) and chicken shawarma (a Middle-Eastern dish made of thinly sliced cuts of meat marinated and cooked after stacking in a vertical skewer) from a food joint near the hospital. The index case developed high grade fever and multiple episodes of vomiting after hospital admission. Subsequently over the next 2 days, 19 more cases were admitted with similar illness, of whom 16 were either students or staff of the hospital. Three of the admitted cases reported about 6 other cases with similar illness treated at other health facilities, thus making the total number hospitalized cases to 26. All of them had consumed chicken shawarma from a nearby food outlet.

Of the 26 individuals who sought medical attention in our hospital and elsewhere, 17 were male and 9 were female. Their median age was 22 (18–25) years. The median (IQR) incubation period of symptom onset was 9.5 (8–12) hours.

Apart from the index case and his co-diner who had taken biriyani along with chicken shawarma from the implicated restaurant, the other 24 people had consumed only chicken shawarma. All cases had greenish loose watery diarrhea. Of 26 cases, 23(88.5%) had high grade fever and vomiting and 25(96.1%) had abdominal pain. Of the 20 cases admitted at our center, 3 required intensive care unit admission because of severe dehydration. All admitted patients recovered completely and were discharged home.

### Isolation of NTS

Microscopic examination of the stool samples could be done for 14 affected individuals. In 13 individuals it revealed pus cells without any ova or cysts. In 10 patients, the stool culture revealed black colonies, which were identified as subsp. *enterica* serovar Enteritidis. *Salmonella* Enteritidis was also isolated from the shawarma sample. Stool PCR was negative in all 14 cases.

The case-control study involving undergraduate students identified 7 more cases of GI illness (not requiring hospitalization), thus taking the total number of cases to 33. Among the 33 cases, 26 had consumed food from the particular food outlet, 4 had consumed food from other outlets, and 3 did not report eating out ( Table 1). Consumption of food from the implicated outlet was significantly associated with GI illness (odds ratio 121.8 [95% CI 28.4 to 522.7]; *P* < 0.001); 26 of 27 persons who had consumed chicken shawarma from that outlet developed GI illness. Applying the Bradford Hill’s criteria, the observed association was deemed to be causally linked; only the criterion of biological gradient was not fulfilled ( Table 2).

**Table 1.** Association of gastrointestinal (GI) illness with varying levels of exposure among cases and controls.

	GI illness present	GI illness absent	Odds ratio (95% CI)	*P*-value
**Level 1**				
Ate food from outside	30	79	29.9 (8.9–100.6)	<0.001
Did not eat food from outside	3	236		
**Level 2**				
Ate food from the suspected food outlet	26	4	121.8 (28.4–522.7)	<0.001
Did not eat food from the suspected food outlet	4	75		
**Level 3**				
Ate chicken shawarma from suspected food outlet	26	1	123.7 (4.2–3665.8)	<0.001
Did not eat chicken shawarma from suspected food outlet	0	3		

**Table 2.** Causality assessment using the Bradford Hill’s criteria.

Criterion	Analogous question in foodborne outbreaks ^10^^,^^11^	Applicability of criterion
Analogy	Do organisms with similar characteristics cause disease related to food consumption under similar condition?	NTS are prevalent in food animals such as poultry, pigs, and cattle as well as in birds and thus can contaminate poorly processed or cooked meat including chicken.
Biological gradient	Is the occurrence of disease related to the amount of food consumed?	No
Coherence	Does information on food quality conflict with epidemiological evidence?	No. The chicken shawarma was proven to be contaminated by NTS. Epidemiologically a link was firmly established between the consumption of chicken shawarma and the illness.
Consistency	Have there been previous reports of disease associated with consumption of this or a similar food?	Yes ^4^
Experiment	Do attempts to improve quality of food (including withdrawal of contaminated product) reduce the occurrence of disease?	Once the outlet was closed, no further cases were reported.
Plausibility	Is the implicated organism likely to survive the food process?	Salmonellae are usually killed at temperatures > 70°C. However, the meat could be undercooked or contaminated after cooking process.
Specificity	Are other sources responsible for any of the disease such as person-to-person or zoonotic transmission?	Unlikely, since 24 of 26 affected individuals had consumed only chicken shawarma.
Strength of association	Are the numbers of people with and without the disease sufficient to prove an association?	Yes
Temporality	Does the occurrence of disease correspond with the known incubation periods?	Yes, NTS infection has an incubation period of 6-72 hours.

### Odds of exposure to shawarma in affected individuals

The data for the case-control study involving undergraduate students was collected between July 25 and August 2, 2019. Of the 348 students who responded, 202 were directly interviewed while 146 had filled up the web-based form. This exercise identified 7 more cases of GI illness (not requiring hospitalization), thus taking the total number of cases to 33. Among the 33 cases, 26 had consumed food from the particular food outlet, 4 had consumed food from other outlets, and 3 did not report eating out ( Table 1). Consumption of food from the implicated outlet was significantly associated with GI illness (odds ratio 121.8 [95% CI 28.41 to 522.66]; P < 0.001); 26 of 27 persons who had consumed chicken shawarma from that outlet developed GI illness. Applying the Bradford Hill’s criteria, the observed association was deemed to be causally linked; only the criterion of biological gradient was not fulfilled ( Table 2).

## Discussion

We found that the outbreak of gastroenteritis caused by *Salmonella* Enteritidis was epidemiologically linked to the consumption of contaminated chicken shawarma from a particular food outlet. Gastroenteritis outbreaks caused by NTS have been previously reported from India and other countries. ^12– 14^ Poultry meat contamination by NTS is also reported. ^15^ Importantly, a study from Jordan found high rates of contamination of chicken meat used in shawarma by *Salmonella* spp. ^16^ Previously, an NTS ( *Salmonella* Thompson) outbreak caused by consumption of chicken shawarma was reported from Canada. ^3^ Microbial contamination of shawarma can occur during the storage, cooking and serving of the meat. Generally, NTS does not survive high temperatures when the cooking process is adequate. However, the important step of secondary cooking of cut slices of meat might be overlooked when the food outlet becomes busy. Such mishaps could be avoided only by ensuring that food safety guidelines are in place in every country and they are strictly enforced.

Two important steps helped in quick containment of the outbreak in our setting: early identification of the contaminated food source and timely intimation of food safety authorities for prohibitory action. Also, since the contaminated food sample was procured while it was still on sale, we could isolate NTS from the source. Moreover, we demonstrated the epidemiological link by performing a case-control study.

One possible limitation of our investigation was that we could not obtain specimens for microbiological testing from the food handlers and the water used for cooking could also not be tested. Notwithstanding, our report helps to highlight shawarma as a potential source of food poisoning.

## Conclusion

In conclusion, epidemiological investigation of foodborne outbreaks could yield important information. Chicken shawarma is a potential source of food poisoning due to NTS. It is important that food safety authorities enforce guidelines for safe preparation and sale of shawarmas and similar products.

## Data availability

### Underlying data

Figshare: Foodborne disease outbreak version 2. https://doi.org/10.6084/m9.figshare.15022065.v2. ^17^

This project contains the following underlying data:

Data file 1. Deepanjali salmonella data (1).xlsx (Foodborne disease outbreak version 2)

Data are available under the terms of the Creative Commons Attribution 4.0 International license (CC-BY 4.0).

## Consent

The data represented in the manuscript was collected as part of an outbreak investigation. An exemption from review was granted by Institute Ethics Committee.
