# Supplementary material for: An outbreak of Salmonella Enteritidis food poisoning following consumption of chicken shawarma: A brief epidemiological investigation
Source: F1000Res. 2022 Mar 28;10:851. Originally published 2021 Aug 25. [Version 4] doi: 10.12688/f1000research.54410.4 (PMC8762677; doi:10.12688/f1000research.54410.4)
Supplement: Peer review history

# Peer review: Reviewer response for version 4

**DOI:** 10.5256/f1000research.120347.r129069

**Published:** 2022-03-29

**Authors:** Samuel Kariuki

**Affiliations:** 1 Centre for Microbiology Research, Kenya Medical Research Institute, Nairobi, Kenya

In light of the revisions done the manuscript now looks much improved and I have no hesitation to recommend it for indexing.

Is the work clearly and accurately presented and does it cite the current literature?

Yes

If applicable, is the statistical analysis and its interpretation appropriate?

Yes

Are all the source data underlying the results available to ensure full reproducibility?

Yes

Is the study design appropriate and is the work technically sound?

Yes

Are the conclusions drawn adequately supported by the results?

Partly

Are sufficient details of methods and analysis provided to allow replication by others?

Yes

Reviewer Expertise:

Epidemiology and genomics of enteric pathogens and AMR

I confirm that I have read this submission and believe that I have an appropriate level of expertise to confirm that it is of an acceptable scientific standard.

# Peer review: Reviewer response for version 3

**DOI:** 10.5256/f1000research.81408.r118746

**Published:** 2022-01-14

**Authors:** Samuel Kariuki

**Affiliations:** 1 Centre for Microbiology Research, Kenya Medical Research Institute, Nairobi, Kenya

This article provides data from an investigation of a *Salmonella Enteritidis* foodborne outbreak due to consumption of contaminated chicken Shawarma. The authors used a case-control approach to perform both an epidemiological and a microbiological investigation to reach a conclusion implicating the consumption of the chicken shawarma as major cause of the GI illness among staff and students at a medical college in India.

 The approach is well articulated and the epi questions are adequate for this kind of investigation. Also the culture and microscopy of stool specimens to identify causal microorganisms was done according to routine procedure. 

 What I need to get clarification on is the result part stating; " *Stool PCR was negative in all 14 cases.". *How was a stool sample that was positive in culture negative by PCR? Was this a case of use of wrong PCR primers?

 The discussion is well articulated and dwells principally on the outcome of the study.

 The first sentence can not be said to be a conclusion from the study and should be deleted - " *epidemiological investigation of foodborne outbreaks could yield important information."*

Is the work clearly and accurately presented and does it cite the current literature?

Yes

If applicable, is the statistical analysis and its interpretation appropriate?

Yes

Are all the source data underlying the results available to ensure full reproducibility?

Yes

Is the study design appropriate and is the work technically sound?

Yes

Are the conclusions drawn adequately supported by the results?

Partly

Are sufficient details of methods and analysis provided to allow replication by others?

Yes

Reviewer Expertise:

Epidemiology ad genomics of enteric pathogens and AMR

I confirm that I have read this submission and believe that I have an appropriate level of expertise to confirm that it is of an acceptable scientific standard.

# Response

**Published:** 2022-01-15

**Authors:** Surendran Deepanjali

**Affiliations:** Jawaharlal Institute of Postgraduate Medical Education and Research (JIPMER), India

We thank the reviewer for the kind appraisal of our manuscript and the valuable comments. In the new version of the manuscript we have made the suggested changes. In the Results section, the sentence "Stool PCR was negative in all 14 cases" has been modified as "Stool PCR was negative  for diarrheagenic *E. coli* and *Campylobacter* spp in all 14 cases.". The first sentence in Conclusions section has been removed. We hope the reviewer find the changes satisfactory.

# Peer review: Reviewer response for version 3

**DOI:** 10.5256/f1000research.81408.r115452

**Published:** 2021-12-22

**Authors:** Veronica Cibin, Marta Leati

**Affiliations:** 1 Istituto Zooprofilattico Sperimentale delle Venezie, Legnaro, Italy

Thank you very much for taking into considerations our suggestions. Now the paper seems to us fluent and clear.

Is the work clearly and accurately presented and does it cite the current literature?

Partly

If applicable, is the statistical analysis and its interpretation appropriate?

I cannot comment. A qualified statistician is required.

Are all the source data underlying the results available to ensure full reproducibility?

Partly

Is the study design appropriate and is the work technically sound?

Yes

Are the conclusions drawn adequately supported by the results?

Partly

Are sufficient details of methods and analysis provided to allow replication by others?

Partly

Reviewer Expertise:

Collection and management of data on zoonoses and zoonotic agents with focus on salmonella; epidemiology of zoonotic agents; food safety; isolation/characterization of zoonotic agents

We confirm that we have read this submission and believe that we have an appropriate level of expertise to confirm that it is of an acceptable scientific standard.

# Response

**Published:** 2021-12-23

**Authors:** Surendran Deepanjali

**Affiliations:** Jawaharlal Institute of Postgraduate Medical Education and Research (JIPMER), India

We thank the respected reviewers for their kind response and would like to put on record that their suggestions helped to substantially enhance the readability of our manuscript.

# Peer review: Reviewer response for version 2

**DOI:** 10.5256/f1000research.79890.r101985

**Published:** 2021-12-10

**Authors:** Veronica Cibin, Marta Leati

**Affiliations:** 1 Istituto Zooprofilattico Sperimentale delle Venezie, Legnaro, Italy

We appreciate the efforts of the authors to modify the paper according to our comments/suggestions; now the manuscript is better organized and the flow of the events is clearer.

 In our opinion, there are some other aspects that are critical (we can see now them in a more evident way after this revision) and we suggest the authors take into account these further comments in order to ameliorate the paper.

 Methods:

- The title “Setting” is not congruent with the content of the paragraph. Moreover, the authors could consider combining the “Setting” paragraph and the “Case-control study” since this last one is defined as “part of the outbreak investigation”, which in fact is the focus of the “Setting” paragraph. Authors could also consider organizing the “Methods” into two main paragraphs: epidemiological investigation and microbiological investigation and then organizing the results in the same way.

Unclear sentences:

- Paragraph ”Recognition of the outbreak...”: “The clinical history of those…to share the chicken shawarma meal”. There is something wrong here with the syntax.
- Paragraph “Case-control study”: “We compared…..at 3 levels”. There is something wrong here with the syntax.

Is the work clearly and accurately presented and does it cite the current literature?

Partly

If applicable, is the statistical analysis and its interpretation appropriate?

I cannot comment. A qualified statistician is required.

Are all the source data underlying the results available to ensure full reproducibility?

Partly

Is the study design appropriate and is the work technically sound?

Yes

Are the conclusions drawn adequately supported by the results?

Partly

Are sufficient details of methods and analysis provided to allow replication by others?

Partly

Reviewer Expertise:

Collocation and management of data on zoonoses and zoonotic agents with focus on salmonella; epidemiology of zoonotic agents; food safety; isolation/characterization of zoonotic agents

We confirm that we have read this submission and believe that we have an appropriate level of expertise to confirm that it is of an acceptable scientific standard, however we have significant reservations, as outlined above.

# Response

**Published:** 2021-12-11

**Authors:** Surendran Deepanjali

**Affiliations:** Jawaharlal Institute of Postgraduate Medical Education and Research (JIPMER), India

We thank the reviewers for reviewing our manuscript again and offering their suggestions. Please find below a point-by-point response to the comments. We hope that reviewers would find the modifications satisfactory.

**Comment:** We appreciate the efforts of the authors to modify the paper according to our comments/suggestions; now the manuscript is better organized and the flow of the events is clearer. In our opinion, there are some other aspects that are critical (we can see now them in a more evident way after this revision) and we suggest the authors take into account these further comments in order to ameliorate the paper.

**Response:** We again thank the reviewers for their kind appraisal of the modifications done in version 2 of the manuscript.

**Comment:** Methods: The title “Setting” is not congruent with the content of the paragraph. Moreover, the authors could consider combining the “Setting” paragraph and the “Case-control study” since this last one is defined as “part of the outbreak investigation”, which in fact is the focus of the “Setting” paragraph. Authors could also consider organizing the “Methods” into two main paragraphs: epidemiological investigation and microbiological investigation and then organizing the results in the same way.

**Response:** We have now divided “Methods” section as Epidemiological investigations and Microbiological investigations. We have now merged the case-control study with the epidemiological investigations.

**Comment: **Unclear sentences: Paragraph ”Recognition of the outbreak...”: “The clinical history of those…to share the chicken shawarma meal”. There is something wrong here with the syntax.

**Response:** As rightly pointed out by the reviewers, this sentence was not syntactically meaningful. Since the information conveyed is not an essential aspect of methodology, we have omitted this sentence in the new version.**

**Comment:** Paragraph “Case-control study”: “We compared…..at 3 levels”. There is something wrong here with the syntax.

**Response:** We have now re-phrased a number of ideas in this paragraph so that the meaning content is clearer. Once again, we hope the reviewers find the modifications made satisfactory.

# Peer review: Reviewer response for version 1

**DOI:** 10.5256/f1000research.57893.r96979

**Published:** 2021-11-04

**Authors:** Veronica Cibin, Marta Leati

**Affiliations:** 1 Istituto Zooprofilattico Sperimentale delle Venezie, Legnaro, Italy

The paper describes a food borne outbreak occurred in India focusing the attention on the epidemiological investigation that was carried out during the two months after the episode.

 The paper is valuable since it contributes to provide data on potential sources of food borne diseases; it was in fact possible to identify the source of infection, the zoonotic agent and the setting. Moreover the authors provide a good selection of the current literature.

 The language is simple and immediate, but the way the contents are organized is chaotic and this makes the text not fluent or fully understandable. For example, in the paragraph “methods”, some results are described, while in the section “results”, in the paragraph “Stool examination”, the microscopic examination of samples is reported and this refers to a method.

 It seems the authors did a very good “in the field” job but the way they describe it is not clear enough. The main issue is that it is not clear whether the outbreak investigation was performed as a learning tool (an exercise?) or if it was needed to identify the source of infection and thus to implement the sanitary measures to avoid additional cases.

 In the section “stool examination” it seems that a sample of shawarma was analysed (but this matrix is not congruent with the “title”) as well and resulted to be contaminated with *Salmonella* Enteritidis but it is not clear when this analysis was performed. This result, with the information obtained from the hospitalized cases, was sufficient to confirm the source of infection; further investigations were probably not needed.

 Additionally, the flow of the diagnostic approach (section “confirmation of cases for NTS infection”) is not clear. What was done as first analysis? With which purpose? Is there a sort of protocol to be followed in order to exclude step-by-step the potential hazards?

 As far as the serotyping, it is not clear which was the method performed.

 Below some other suggestions:

- Abstract: “NTE” is used for the first time, please explain this abbreviation.
- Table 2 ”Coherence”: it has to be clarified the link between the question asked and the applicability of the criterion. 
- Case control study: we suggest here to use the original reference when you cite the Bradford hill guidelines.
- Conclusion: it is quite poor and generic; authors could add information on possible strategies in order to avoid similar outbreaks in the future. For example, the consideration “it is important that food…similar products.” of the discussion could be moved here.

Is the work clearly and accurately presented and does it cite the current literature?

Partly

If applicable, is the statistical analysis and its interpretation appropriate?

I cannot comment. A qualified statistician is required.

Are all the source data underlying the results available to ensure full reproducibility?

Partly

Is the study design appropriate and is the work technically sound?

Yes

Are the conclusions drawn adequately supported by the results?

Partly

Are sufficient details of methods and analysis provided to allow replication by others?

Partly

Reviewer Expertise:

Collocation and management of data on zoonoses and zoonotic agents with focus on salmonella; epidemiology od zoonotic agents; food safety; isolation/characterization of zoonotic agents

We confirm that we have read this submission and believe that we have an appropriate level of expertise to confirm that it is of an acceptable scientific standard, however we have significant reservations, as outlined above.

# Response

**Published:** 2021-11-28

**Authors:** Surendran Deepanjali

**Affiliations:** Jawaharlal Institute of Postgraduate Medical Education and Research (JIPMER), India

We thank the reviewers for their kind appraisal of our manuscript and their constructive comments. Please find below a point-by-point response to the comments. We hope that reviewers find the changes satisfactory. We will be happy to respond to any further comments or suggestions by the reviewers.

 Comment: *The paper describes a food borne outbreak occurred in India focusing the attention on the epidemiological investigation that was carried out during the two months after the episode. The paper is valuable since it contributes to provide data on potential sources of food borne diseases; it was in fact possible to identify the source of infection, the zoonotic agent and the setting. Moreover, the authors provide a good selection of the current literature.*

 Response: We thank the reviewers for their encouraging words.

 Comment: *The language is simple and immediate, but the way the contents are organized is chaotic and this makes the text not fluent or fully understandable. For example, in the paragraph “methods”, some results are described, while in the section “results”, in the paragraph “Stool examination”, the microscopic examination of samples is reported and this refers to a method.*

 Response: We have now moved all the methodological aspects to the Methods section. The findings through clinical, microbiological and the epidemiological study have been moved completely to the Results section. We have also renamed the section “confirmation of cases of NTS infection” as “Microbiological investigations” for better clarity.

 Comment: *It seems the authors did a very good “in the field” job but the way they describe it is not clear enough. The main issue is that it is not clear whether the outbreak investigation was performed as a learning tool (an exercise?) or if it was needed to identify the source of infection and thus to implement the sanitary measures to avoid additional cases.*

 Response: To avoid this ambiguity we have now added further clarification in the Settings section - “The data presented in the study was collected as part of an outbreak investigation which was a public health exercise to identify the source of infection and taking steps to prevent further infections.”

 Comment: *In the section “stool examination” it seems that a sample of shawarma was analysed (but this matrix is not congruent with the “title”) as well and resulted to be contaminated with Salmonella Enteritidis but it is not clear when this analysis was performed. This result, with the information obtained from the hospitalized cases, was sufficient to confirm the source of infection; further investigations were probably not needed.*

 Response: The section ‘stool examination” is renamed as “Isolation of NTS”. The analysis of shawarma sample was done on the same day when the outbreak was identified and this is now made clear in the section “Microbiological investigations”.

 Comment: *Additionally, the flow of the diagnostic approach (section “confirmation of cases for NTS infection”) is not clear. What was done as first analysis? With which purpose? Is there a sort of protocol to be followed in order to exclude step-by-step the potential hazards? As far as the serotyping, it is not clear which was the method performed.*

 The first step was the identification of a possible foodborne disease outbreak which was suspected when many cases with gastrointestinal illness was admitted after consumption of the same food item from a single outlet. An investigation to confirm the contaminated source was done as part of the public health exercise. The subsequent case control study was undertaken to ascertain that the clinically observed association is indeed true. Although no pre-specified protocol was followed, the case-control study was envisaged by the Head of our institution to make sure that the observed association of the illness with shawarma consumption was a scientifically valid one. The method used for serotyping is now made clear in the section “Microbiological investigations”.

 Comment: *Abstract: “NTE” is used for the first time, please explain this abbreviation.*

 Response: We thank the reviewers for pointing this out. The suggested change has been made.

 Comment: *Table 2 ”Coherence”: it has to be clarified the link between the question asked and the applicability of the criterion.*

 Response: We have revised the explanation provided for this criterion in Table2.

 Comment: *Case control study: we suggest here to use the original reference when you cite the Bradford hill guidelines.*

 Response: The original reference has been added now.

 Comment: *Conclusion: it is quite poor and generic; authors could add information on possible strategies in order to avoid similar outbreaks in the future. For example, the consideration “it is important that food…similar products.” of the discussion could be moved here.*

 Response: As suggested by the reviewers, we have moved the point about need for food safety guidelines to the Conclusions section.
